# Inhibition of glucose turnover by 3-bromopyruvate counteracts pancreatic cancer stem cell features and sensitizes cells to gemcitabine

**DOI:** 10.18632/oncotarget.2120

**Published:** 2014-06-15

**Authors:** Orkhan Isayev, Vanessa Rausch, Nathalie Bauer, Li Liu, Pei Fan, Yiyao Zhang, Jury Gladkich, Clifford C. Nwaeburu, Jürgen Mattern, Martin Mollenhauer, Felix Rückert, Sebastian Zach, Uwe Haberkorn, Wolfgang Gross, Frank Schönsiegel, Alexandr V. Bazhin, Ingrid Herr

**Affiliations:** ^1^ Molecular OncoSurgery, University of Heidelberg and German Cancer Research Center (DKFZ), Heidelberg, Germany; ^2^ General-, Visceral- & Transplantation Surgery, Section Experimental Surgery, University of Heidelberg, Germany; ^3^ Department of Surgery, University Hospital Mannheim, Germany; ^4^ Department of Nuclear Medicine, University of Heidelberg, Germany; ^5^ Department of General-, Visceral-, Transplantation-, Vascular- and Thoraxsurgery, University Hospital München - Großhadern, Germany

**Keywords:** Pancreatic cancer, Cancer stem cells, Novel therapeutics, Warburg effect, 3-Bromopyruvate

## Abstract

According to the cancer stem cell (CSC) hypothesis, the aggressive growth and early metastasis of pancreatic ductal adenocarcinoma (PDA) is due to the activity of CSCs, which are not targeted by current therapies. Otto Warburg suggested that the growth of cancer cells is driven by a high glucose metabolism. Here, we investigated whether glycolysis inhibition targets CSCs and thus may enhance therapeutic efficacy. Four established and 3 primary PDA cell lines, non-malignant cells, and 3 patient-tumor-derived CSC-enriched spheroidal cultures were analyzed by glucose turnover measurements, MTT and ATP assays, flow cytometry of ALDH1 activity and annexin positivity, colony and spheroid formation, western blotting, electrophoretic mobility shift assay, xenotransplantation, and immunohistochemistry. The effect of siRNA-mediated inhibition of LDH-A and LDH-B was also investigated. The PDA cells exhibited a high glucose metabolism, and glucose withdrawal or LDH inhibition by siRNA prevented growth and colony formation. Treatment with the anti-glycolytic agent 3-bromopyruvate almost completely blocked cell viability, self-renewal potential, NF-κB binding activity, and stem cell-related signaling and reverted gemcitabine resistance. 3-bromopyruvate was less effective in weakly malignant PDA cells and did not affect non-malignant cells, predicting minimal side effects. 3-bromopyruvate inhibited *in vivo* tumor engraftment and growth on chicken eggs and mice and enhanced the efficacy of gemcitabine by influencing the expression of markers of proliferation, apoptosis, self-renewal, and metastasis. Most importantly, primary CSC-enriched spheroidal cultures were eliminated by 3-bromopyruvate. These findings propose that CSCs may be specifically dependent on a high glucose turnover and suggest 3-bromopyruvate for therapeutic intervention.

## INTRODUCTION

Pancreatic ductal adenocarcinoma (PDA) is one of the most aggressive malignancies and is typically diagnosed in an advanced state, with extensive local invasion, early systemic dissemination, and marked resistance to chemo- and radiotherapies [1]. Despite the low response rate to current treatment options and the modest overall survival benefit and rapid development of resistance, gemcitabine has been adopted as the standard therapy for advanced pancreatic cancer [2]. However, neither gemcitabine nor other chemotherapies, such as FOLFIRINOX, directly target the pathways responsible for the aggressive growth, early metastasis, and therapy resistance of PDA.

Markers for cancer stem cells (CSCs), including c-Met, CD133, CD44, CxCR4, ALDH1, and Sox2, have been identified in PDA and are thought to be responsible for the aggressive growth, early metastasis, high resistance to therapy, and frequent relapse despite surgery and chemotherapy [3-6]. CSCs exhibit the properties of normal stem cells, such as chemotherapy resistance, high DNA repair capacity, apoptosis resistance, and self-renewal potential, with the latter being characterized by the ability to form spheroids and colonies and to recapitulate a tumor after xenotransplantation. In addition, increased activity of the transcription factor NF-κB contributes to the aggressive nature of pancreatic CSCs [7].

Nearly 100 years ago, Otto Warburg described the enhanced glucose metabolism and production of lactate by cancer cells under aerobic conditions, with mitochondrial energy recovery being strongly inhibited [8]. Metabolic reactions in the glycolytic pathway are catalyzed by such enzymes as hexokinase II (HKII), glycerinaldehyd-3-phosphat-dehydrogenase (GAPDH), and lactate dehydrogenase (LDH), and data from proteomic analyses demonstrate the upregulation of glycolytic enzymes in pancreatic cancer [9]. Today, positron emission tomography (PET) utilizes Warburg's findings to detect tumors based on the accumulation of fluorescence-marked glucose. A higher PET signal correlates to an advanced tumor stage, and experimental studies demonstrate that increased glucose uptake and lactate production occur particularly in late tumorigenesis [10-13]. Because CSCs are enriched after several treatment cycles and in metastasis during late tumorigenesis [6, 14], these data propose that CSCs may be specifically dependent on a high glucose turnover; nonetheless, this assumption has not been investigated to date.

The cancer cell-specific glycolysis inhibitor 3-bromopyruvate (3BrP), a halogenated analog of pyruvate [15], inhibits HKII [16] and GAPDH [17] and mitochondrial ATP production [16], which leads to the depletion of ATP resources within minutes, particularly in cells with mitochondrial DNA deletion and respiration defects, and induces massive cell death [18]. In addition, 3BrP acts as an alkylating agent, which enhances tumor cell death [18]. The anti-tumor effects of 3BrP have been demonstrated in PDA cell lines *in vitro*, in subcutaneous xenografts [19], and in orthotopically transplanted mouse xenografts [20]. Recently, 3BrP was first tested in a case study at University Hospital Frankfurt, Germany, on a 16-year-old patient with advanced metabolically active fibrolamellar hepatocellular carcinoma [21]; unsuccessful therapeutic intervention with chemotherapy and sorafenib had been first applied. The conclusions were that tumor necrosis was more extensive than after the application of known cytostatic drugs, liver regeneration was not inhibited, and the patient was able to survive longer than expected with an improved quality of life [21]. These data point to the possibility that 3BrP targets CSCs, which are made responsible for aggressive growth.

In the present study we analyzed, whether 3BrP targets pancreatic CSC features and sensitizes therapy-resistant PDA cells to gemcitabine. Through the use of a broad panel of established and primary PDA cells and *in vitro* and *in vivo* models, we demonstrate that 3BrP targets the CSC self-renewal potential and inhibits several stem cell-related factors. Our data suggest that 3BrP efficiently eliminates highly aggressive PDA cells and, together with gemcitabine, leads to complete tumor elimination with low side effects.

## RESULTS

### Survival of PDA cells with CSC features depends on glucose turnover

**Figure 1:**
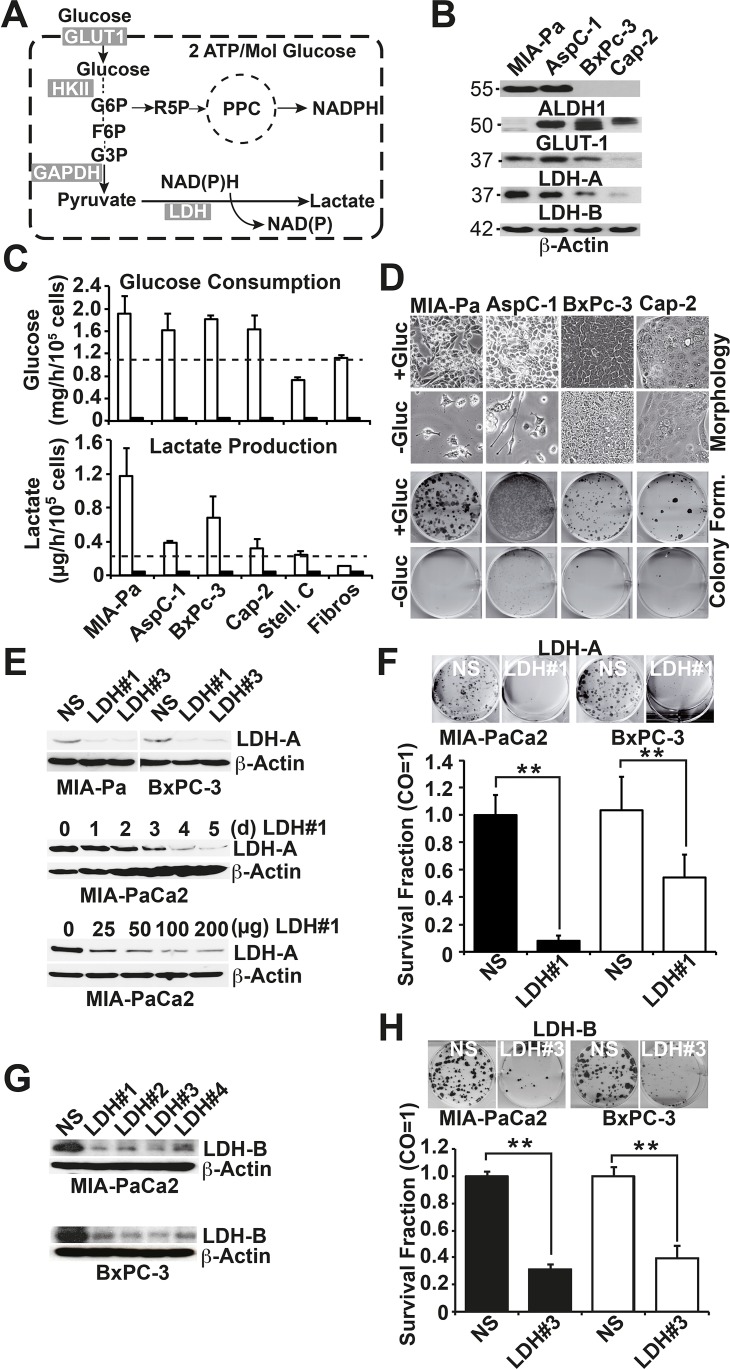
Survival of pancreatic cancer stem-like cells depends on glucose turnover (A) Schematic of glycolysis. GLUT1, glucose transporter 1; HKI, hexokinase II, GAPDH, glyceraldehyde 3-phosphate dehydrogenase; LDH, lactate dehydrogenase; G6P, glucose-6-phosphate; R5P, ribose-5-phosphate; PPC, pentose phosphate cycle; F6P, fructose-6-phosphate; G3P, glycerinaldehyde-3-phosphate. (B) Highly aggressive MIA-PaCa2 (MIA-Pa) and AsPC-1 cells and less aggressive BxPc-3 and Capan-2 (Cap-2) cells were regularly cultured in a cell culture medium with 25 mmol/L Glucose for 72 h. The proteins were harvested, and the expression of ALDH1, GLUT1, LDH-A and LDH-B was analyzed by western blotting; β-Actin expression served as a loading control. The size of the proteins is indicated in kilodaltons on the left. (C) Cells were incubated for 24 h in a medium with 25 mmol/L Glucose (white bars) or 0.15 mmol/L (black bars), and the amount of glucose or lactate in the medium was determined using a DRY-CHEM FCD3500 machine. (D) Upper panel: Cells were cultured in a medium with 25 (+Gluc) or 0.15 mmol/L (-Gluc) glucose, and images depicting the cell morphology were captured three weeks later. Alternatively, cells were seeded at a density of 3×10^5^ cells in 6-well tissue culture plates. Lower panel: the cells were trypsinized after culture for 72 h in a medium with 25 (+Gluc) or 0.15 mmol/L (-Gluc) glucose, and 200 MIA-PaCa2 and AsPC-1 or 2000 BxPc-3 or Capan-2 cells/well were seeded in 6-well plates in medium with 25 (+Gluc) or 0.15 mmol/L (-Gluc) glucose. Cells were grown without a change of the medium for two weeks, followed by Coomassie blue staining of the colonies. Images of the fixed colonies were obtained by scanning the plates. (E) Upper panel: Cells were treated with non-specific siRNA (NS) or 2 specific siRNAs directed against LDH-A (LDH#1, LDH#3) for 3 days at a concentration of 50 pmol; proteins were then harvested. Middle panel: Cells were treated with 50 pmol siRNA targeting LDH#1; proteins were harvested at 0, 1, 2, 3, 4, or 5 days (d) after transfection. Lower panel: Cells were treated with 25, 50, 100, or 200 pmol siRNA targeting LDH#1, and the proteins were harvested 3 days later. Expression of LDH-A was analyzed by western blotting. (F) Cells were transfected with 100 pmol non-specific siRNA (NS siRNA) or with a specific siRNA directed against LDH-A (LDH#1 siRNA) and the colony formation was evaluated as described above (G) Cells were treated with non-specific siRNA (NS) or 4 specific siRNAs directed against LDH-B for 3 days at a concentration of 100 pmol followed by western blot analysis. (H) Cells were transfected with 100 pmol non-specific siRNA (NS siRNA) or with a specific siRNA directed against LDH-B and colony formation was analyzed as described above.

To examine whether pancreatic CSCs are dependent on glycolysis (schematic of glycolysis Fig. 1A), we examined 4 human established PDA cell lines with high or low CSC features (see Fig. S1). A western blot analysis under regular culture conditions with 25 mmol/L glucose revealed that the MIA-PaCa2 and AsPC1 cells expressed high levels of the CSC-marker aldehyde dehydrogenase isoform 1 (ALDH1), whereas the BxPc-3 and Capan-2 cells did not (Fig. 1B). ALDH1 expression correlated to the amount of CSC features, such as the p53 and K-ras status, *in vitro* morphology, self-renewal capacity, gemcitabine resistance, and expression of E-cadherin and vimentin (Fig. S1). Similarly, the glycolytic enzymes LDH-A and LDH-B were highly expressed in the two more aggressive cell lines but to a lower extend in the two less aggressive cell lines, whereas the amount of GLUT-1 expression could not be correlated to the level of CSC features. The culture for 3 days in a medium with a minimal amount of glucose (0.15 mmol/L) was accompanied by a total absence of glucose consumption and lactate production (Fig. 1C). As expected, glucose consumption and lactate production under culture conditions in the presence of glucose were generally lower in non-malignant stellate cells and fibroblasts. However, no obvious difference in glucose turnover was observed between the highly and low aggressive PDA cell lines. In contrast, morphological studies after three weeks of culture in a medium with a minimal amount of glucose (0.15 mmol/L) revealed that most of the highly aggressive MIA-PaCa2 and AsPC-1 cells were dead, whereas most of the less aggressive BxPC-3 and Capan-2 cells survived (Fig. 1D). Similarly, colonies, which are formed by the highly aggressive and CSC-like cells only (Fig. S1), were significantly reduced upon deprivation of glucose to a minimal amount (0.15 mmol/L) in all cell lines (Fig. 1D, Fig. S2A). To further elucidate the function of glucose metabolism in colony formation, we knocked down LDH-A in MIA-PaCa2 and BxPc-3 cells using three different LDH-A-targeting siRNAs. Compared to the non-specific control siRNA, two of the LDH-A siRNAs completely downregulated LDH-A protein expression at a concentration of 50 pmol, and the strongest effect, as evaluated by western blotting, was observed at five days after transfection (Fig. 1E). The inhibition of the LDH-A protein was accompanied by the inhibition of colony formation (Fig. 1F). Likewise, the siRNA-mediated downregulation of LDH-B (Fig. 1G) strongly inhibited colony formation (Fig. 1H), suggesting that both isoforms of LDH are required for survival of CSCs.

### 3-Bromopyruvate overcomes gemcitabine resistance

**Figure 2:**
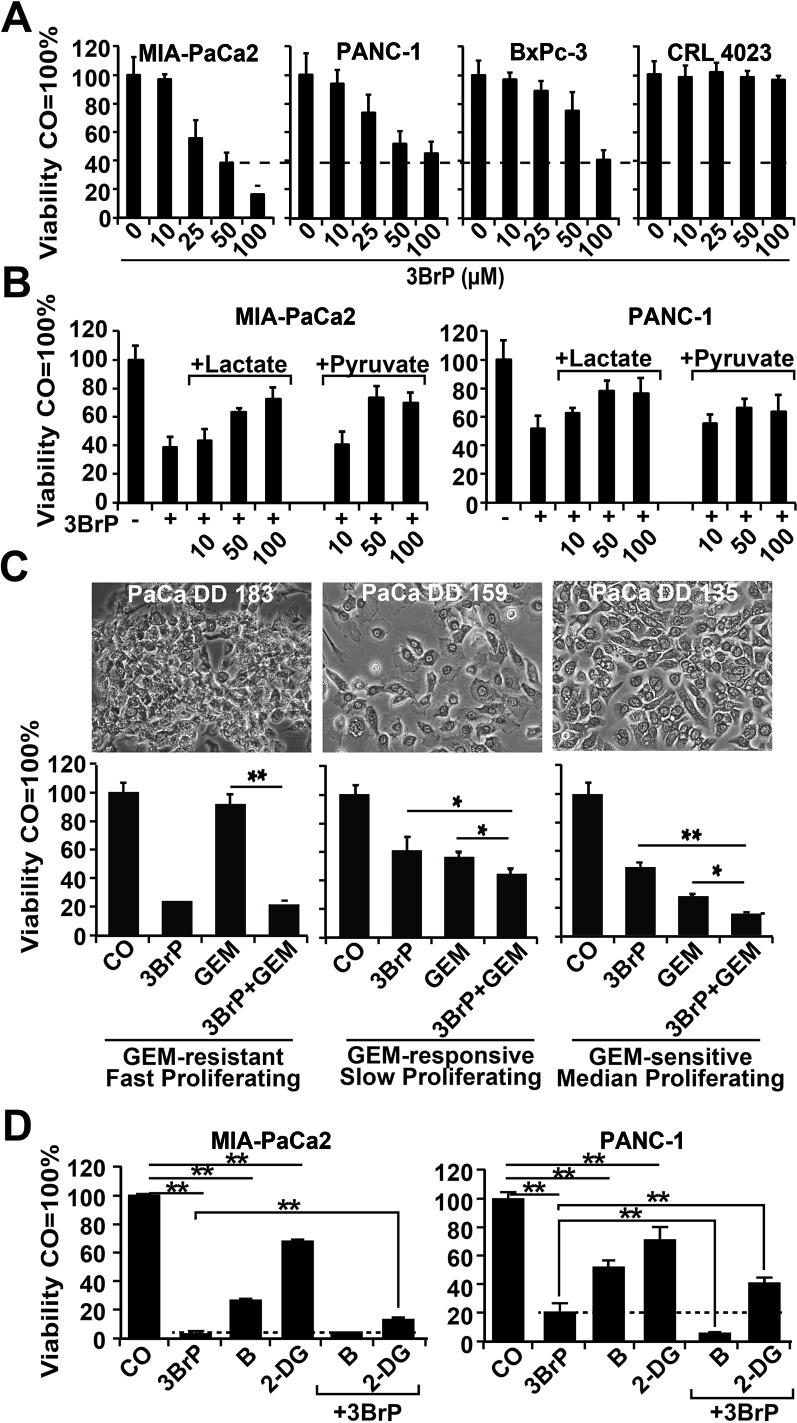
3BrP strongly inhibits viability in highly malignant cells and thereby sensitizes them to gemcitabine (A) Highly malignant MIA-PaCa2 and PANC-1 cells and less malignant BxPc-3 and immortalized pancreatic ductal CRL 4023 cells were treated with 10, 25, 50, and 100 μM 3-bromopyruvate (3BrP). After 24 hours, viability based on the NAD(P)H content was measured by the MTT assay. (B) Cells were left untreated or were treated with 10, 50, or 100 mM lactate or pyruvate for 24 h, followed by treatment with 3BrP (50 μM) for an additional 24 h. (C) Primary PaCa DD 183, PaCa DD 159, and PaCa DD 135 cells were left untreated (CO, GEM) or were pre-treated with 3BrP (50 μM, 3BrP, GEM+3BrP) for 24 h. Afterwards, the cells were treated with gemcitabine (50 nM, GEM, 3BrP+GEM) for an additional 72 h. Cell viability was measured with the Cell Titer Glo-Assay and analyzed as described above. (D) Cells were left untreated (CO) or were pretreated with 3BrP for 24 h, followed by treatment with Bortezomib (B, 100 nM) or 2-deoxy-d-glucose (2-DG, 10 mM) for additional 72 h—either alone, or combined with 3BrP. Ninty-six hours after 3BrP treatment the cell viability based on the ATP content was measured with the Cell Titer Glo-Assay.

Because a therapeutic approach using siRNA is far from clinical application for therapeutic inhibition of LDH, we elucidated the influence of 3BrP on CSC features. This agent is inexpensive and commercially available and has already demonstrated promising results in a patient case study [21]. Cells were treated with 3BrP at concentrations ranging from 10 to 100 μM, and cell viability was examined 24 h later with the MTT assay, which measures the NAD(P)H content [22]. Starting at a dose of 25 μM, 3BrP dose-dependently inhibited viability, with the strongest effects observed in the more aggressive MIA-PaCa2 and PANC-1 cells, whereas the less aggressive BxPc-3 cells were minor affected; no influence at all was observed in the non-malignant CRL 4023 cells (Fig. 2A). These results were due to the specific inhibition of glucose turnover by 3BrP, as competition with the analogs lactate or pyruvate for 24 h and subsequent treatment with 50 μM 3BrP for another 24 h counteracted the 3BrP-induced reduction in viability (Fig. 2B). After pre-treatment of the cells with 3BrP for 24 h, followed by treatment with gemcitabine for an additional 72 h alone or in combination, we found that 3BrP strongly inhibited viability in the three cancer cell lines but not in the non-malignant CRL 4023 cells, as detected by the MTT assay and the Cell Titer Glo-Assay, which measures the ATP content (Fig. S2 B, C). In contrast, gemcitabine only lowered viability in the less aggressive and non-malignant cells, whereas the two more aggressive cell lines were resistant. We then used primary human PDA cell lines to evaluate the effects in additional patient-related primary PDA models. Treatment with 3BrP and gemcitabine alone or in combination revealed that PaCa DD 183 is gemcitabine resistant, PaCa DD 159 is slightly responsive to gemcitabine, and PaCa DD 135 is gemcitabine sensitive (Fig. 2C). In accordance with our results in established PDA cell lines, the gemcitabine-resistant PaCa DD 183 cells were most sensitive to 3BrP in comparison to PaCa DD 159 and PaCa DD 135 cells, which were responsive to 3BrP though to a minor extent. We also tried the combination of 3BrP with 2 other metabolic inhibitors and used the glycolysis inhibitor 2-desoxy-d-glucose (2-DG) and the proteasome inhibitor Bortezomib (B). Each agent significantly inhibited the viability, but the combination with 3BrP was superior (Fig. 2D). Likewise, 2-DG significantly increased the efficacy of gemcitabine (Fig. S2 D), suggesting that inhibition of glycolysis in general enhances the anti-tumor activity. Together, 3BrP largely appears to influence more advanced PDA cells, it overcomes gemcitabine resistance and enhances the therapeutic efficacy of other metabolic inhibitors.

### 3BrP inhibits self-renewal potential and stem cell related signaling

**Figure 3:**
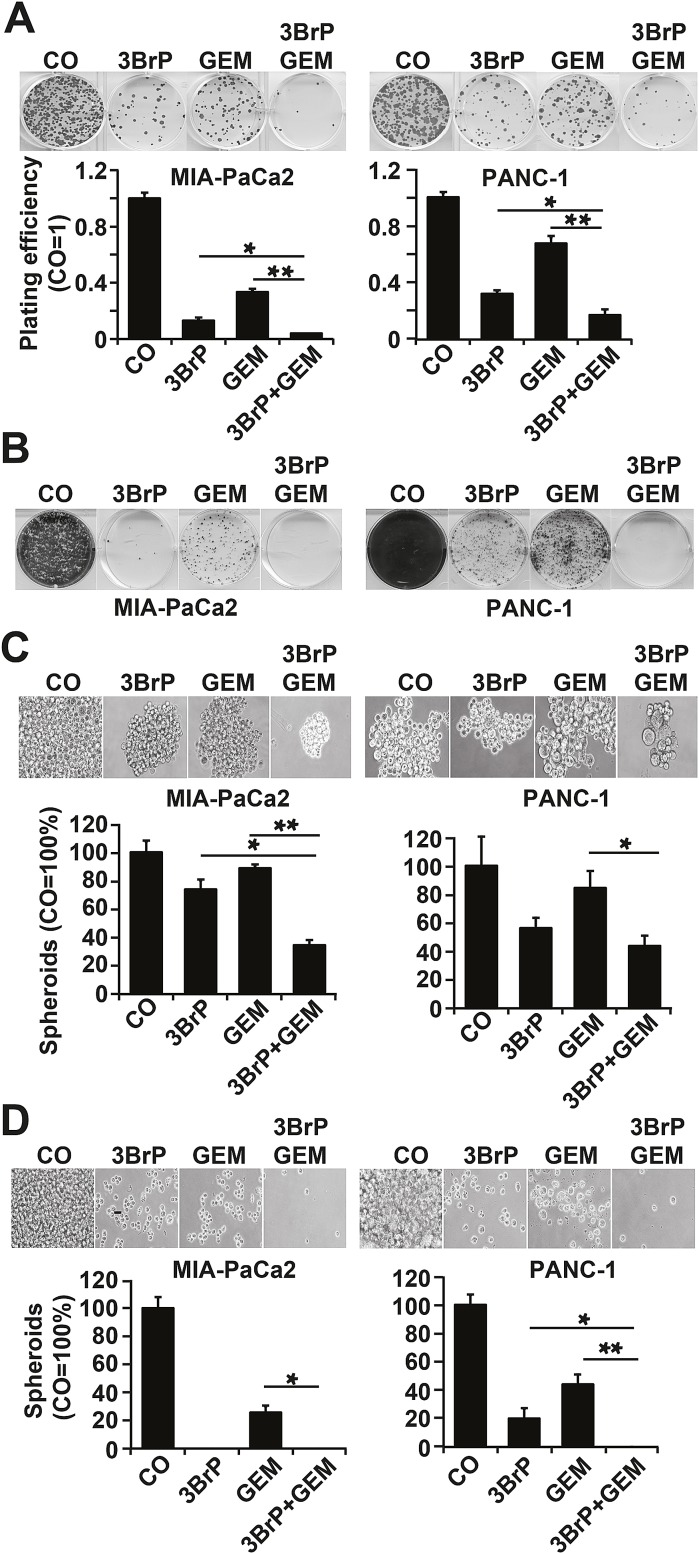
3BrP inhibits colony and spheroid formation (A) Cells were seeded at a density of 1.5 × 10^5^ cells/mL in 6-well plates. After 24 hours, the cells were treated as described in Fig. 2C. Colony formation was analyzed as described in Fig. 1F. (B) 100 cells/mL were seeded in 6-well plates. After colony formation, cells were treated as described above. At 72 hours after gemcitabine treatment, the cells were trypsinized, and 100 viable cells were re-seeded for a second round of treatment under identical conditions. (C) Cells were seeded at 2×10^2^ cells/mL in 12-well low-adhesion plates. Twenty-four hours later the cells were treated as described in Fig. 2C. Spheroid formation was analyzed as described in M&M. For quantification, single-cell suspensions were prepared, and the amount of cells was calculated by counting the trypan blue-negative cells with a Neubauer counting chamber. (D) Cells were seeded at low densities. After formation of spheroids, the cells were treated as described in Fig. 2C. Equal numbers of viable cells from each group were re-seeded for a second round of treatment under identical conditions.

**Figure 4:**
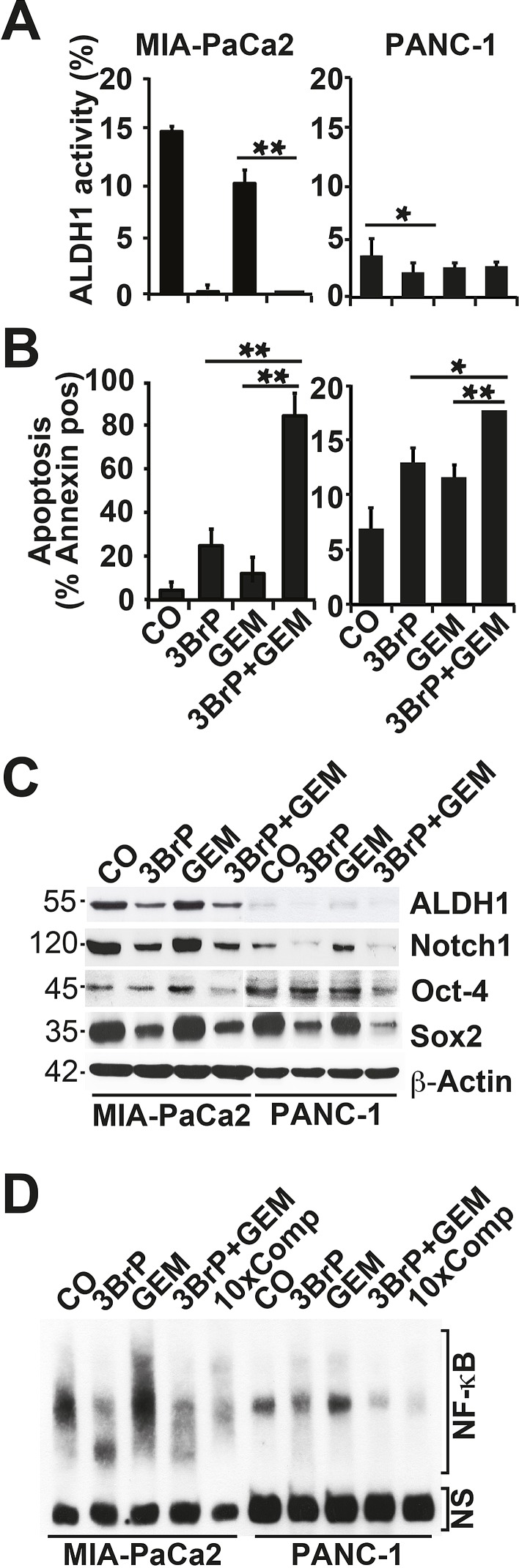
3BrP induces stem cell signaling and apoptosis (A) Cells were treated as described in Fig. 2C. ALDH1 activity was measured with the non-toxic ALDEFLUOR assay kit and FACS analysis. (B) Apoptosis was measured by annexin staining, followed by FACS analysis. (C) Protein expression of ALDH1, Notch 1, Oct-4, and Sox2 was analyzed by western blotting. (D) Nuclear extracts were prepared and DNA binding was analyzed by an electrophoretic mobility shift assay (EMSA) using a specific biotin-labeled oligonucleotide probe for NF-κB. NS marks non-specific DNA binding. 10×Comp: A 10-fold excess of unlabeled oligonucleotide was added to the control DNA-binding reaction performed with nuclear extract from untreated cells and incubation with the biotin-labeled oligonucleotide probe for NF-κB.

To investigate whether 3BrP inhibits the self-renewal potential, cells were seeded at clonal density, followed by treatment 24 h later. 3BrP more strongly inhibited colony formation than gemcitabine did, and both agents together nearly abolished colony formation entirely (Fig. 3A). To evaluate whether a few CSCs might have resisted the treatment and thus may be capable of mediating tumor relapse, we examined the potential of second-generation colony formation. Cells were seeded at clonal density and, after colony formation, the cells were treated. At 72 hours after gemcitabine treatment, the cells were trypsinized and re-seeded at clonal density for a second round of treatment under identical conditions. This resulted in an even stronger prevention of colony formation in both single treatments, whereas 3BrP was more effective than gemcitabine (Fig. 3B). Most importantly, the combination of both agents totally prevented colony formation. Similarly, treatment of spheroidal-growing cells led to a reduction in spheroid sizes: 3BrP was stronger than gemcitabine, and both agents together had an additive effect (Fig. 3C). Repeated treatment of the re-seeded spheroidal growing cells completely prevented the formation of secondary spheroids (Fig. 3D). The observed effects on the self-renewal potential were confirmed by the measurement of the ALDH1 activity in MIA-PaCa2 cells using the ALDEFLUOR assay and FACS analysis, and 3BrP and the combined treatment, but not gemcitabine alone, completely inhibited ALDH1 activity (Fig. 4A). In contrast, the PANC-1 cells already had a basal low ALDH1 activity, which was not further significantly reduced by treatment. However, 3BrP induced stronger apoptosis than gemcitabine, and the combination of both agents further increased the induction of apoptosis in both cell lines, as measured by annexin staining and FACS analysis (Fig. 4B). To examine the influence of 3BrP on stem cell signaling, protein expression was analyzed by western blotting. 3BrP, but not gemcitabine, strongly diminished the expression of ALDH1, Notch1, Oct-4, and Sox2 in both cell lines (Fig. 4C), though the combination therapy had no stronger effect than 3BrP alone. To examine NF-κB activity, we investigated the binding activity of nuclear proteins to the NF-κB consensus-binding site by EMSA. Although 3BrP reduced the binding activity of NF-κB complexes, gemcitabine induced this activity (Fig. 4D). The combination of both agents had no stronger effect than 3BrP alone in MIA-PaCa2 cells, yet it completely abolished NF-κB activity in PANC-1 cells.

### 3BrP reduces tumor engraftment and growth and increases gemcitabine efficacy *in vivo*

**Figure 5:**
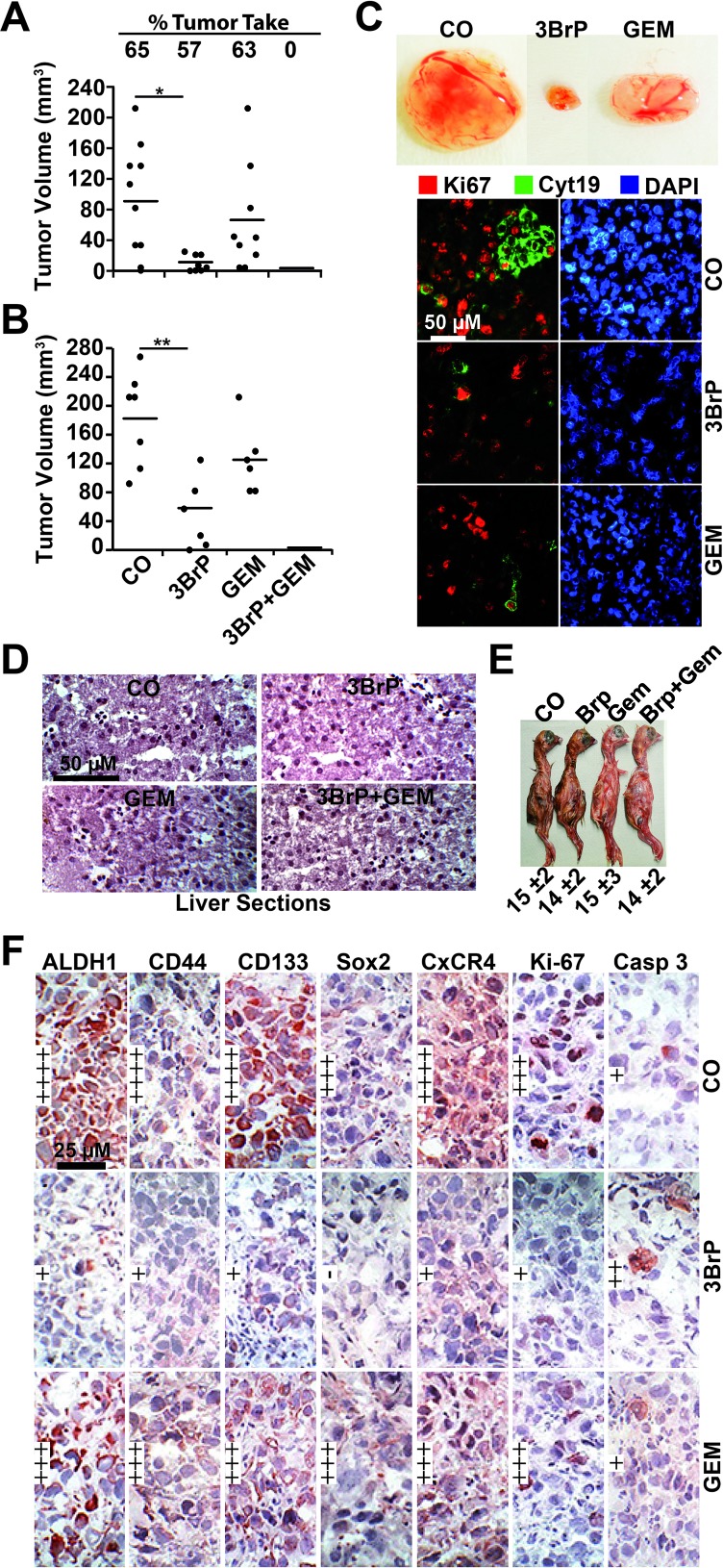
3BrP inhibits tumor growth and inhibits CSC marker expression *in vivo* (A) MIA-PaCa2 cells were treated as described in Fig. 2C, followed by transplantation to fertilized chicken eggs at day 9 of embryonic development, as described in M&M. At day 17, the percentage of engrafted tumors and the volumes of resected tumors were determined. (B) Untreated PANC-1 cells were transplanted into fertilized chicken eggs at day 9 of embryonic development. At day 11, a filter paper was placed next to the CAM, and 10 μl each of PBS (CO), or 3BrP (50 μM) were dropped onto the filter papers. After 24 hours (day 12), 10 μl PBS was dropped onto the filter papers of the control, 3BrP and gemcitabine (GEM 100 nM) groups, whereas 10 μl gemcitabine + 10 μl 3BrP were added to the combination groups. The tumor xenografts were resected at day 17, and the tumor volume was measured as described in M&M. The significance between the single treatments and the combination group could not be determined because the tumor xenografts were eliminated in the combination group; the single data points are therefore 0. (C) Representative images of the tumor sizes of each group of PANC-1 xenografts are shown except of tumors of the combination group, which were completely eliminated. Double-immunofluorescence staining of the xenograft tissue with human-specific antibodies against cytokeratin 19 (Cyt19) and the proliferation marker Ki67 and DAPI staining are shown. (D) At day 17, the embryonal livers were resected and representative H&E stainings are shown. (E) The weight of the embryos was determined at day 17 and is presented as the mean weight ± SD. (F) Frozen xenograft tumor tissue sections from PANC-1 xenografts were analyzed by immunohistochemistry for the expression of the CSC markers ALDH1, CD44, CD133, and Sox2, the invasion marker CxCR4, the proliferation marker Ki-67, and the apoptosis marker “cleaved fragment of active caspase 3”. Representative photographs under 400× magnification are shown. Very high (++++), high (+++), medium (++), low (+), and very low to absent (-) expression is indicated.

**Figure 6:**
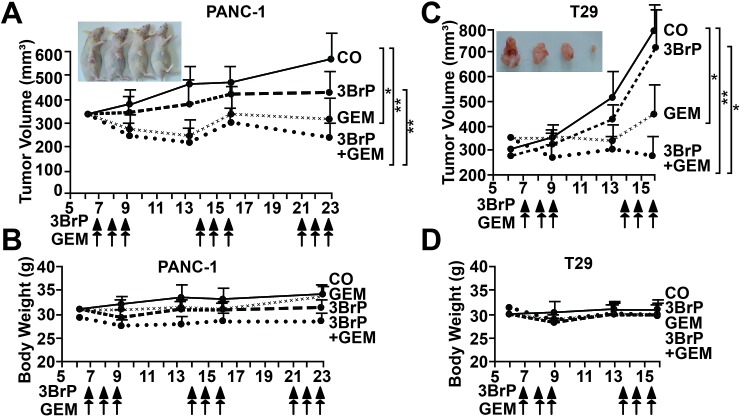
3BrP inhibits tumor growth in mice and increases gemcitabine efficacy (A) PANC-1 cells (1 × 10^6^ in 200 μl cell culture medium) were subcutaneously transplanted into the flanks of 9 mice. Seven days later, after diameters of tumors reached 8-10 mm, the mice were grouped randomly. Mice were treated with PBS (CO), 3BrP (5 mg/kg) and Gemcitabine (GEM, 3 mg/kg) alone, or combined (3BrP+GEM) for 3 consecutive days as indicated by arrows. After a break of 4 days, the mice were treated again for 3 consecutive days. The tumor size was measured as described in M&M throughout the experiment. At day 23 after tumor transplantation the mice were humanely sacrified. (B) The body weight of mice of each group was determined throughout the experiment. (C, D) Primary T29 spheroidal cells were transplanted to mice and mice were treated as described in above.

To evaluate whether 3BrP might inhibit tumor take and growth, we treated MIA-PaCa2 cells *in vitro*, followed by xenotransplantation to the CAM of fertilized chicken eggs. 3BrP reduced the engraftment to 57% compared to 65% in the control group, whereas gemcitabine had no obvious effect, with a rate of 63%. However, the combined treatment totally blocked the engraftment of tumors, suggesting that all CSCs were eliminated (Fig. 5A). Examination of the resected tumors revealed that the volume was strongly reduced in the 3BrP-treated group; gemcitabine was less effective, and the combination treatment completely prevented tumor growth. In a second experiment, we transplanted untreated PANC-1 cells and treated the xenografts *in ovo* [21]. The results confirm the data above (Fig. 5B). These results were underlined by double-immunofluorescence staining of the resected tumor tissue for the proliferation marker Ki67 and a marker of human cells, cytokeratin 19. The expression of these markers was strongly reduced after single treatments, with the most pronounced effects in the 3BrP-treated groups (Fig. 5C). The single or combination treatment was non-toxic *in vivo* as concluded from H&E staining of embryonal liver, where no necrosis was detected (Fig. 5D). Likewise, the embryos of either group looked normal and had a comparable body weight (Fig. 5E). The tumor tissue derived from the PANC-1 xenografts was further analyzed by immunohistochemistry, demonstrating that 3BrP, but not gemcitabine, strongly reduced the expression of the stem cell markers ALDH1, CD44, CD133, Sox2, and CxCR4 and the proliferation marker Ki67, whereas the apoptosis marker “cleaved fragment of active caspase 3” was strongly enhanced (Fig. 5F, Fig. S3). Although the chick embryo model is innovative and cost- and time-effective, we wondered whether 3BrP alone or combined with gemcitabine would also inhibit the growth of human tumor xenografts in mice. PANC-1 and primary T29 PDA cells were subcutaneously transplanted into the flanks of immunodeficient mice. After development of tumors, the mice received intraperitoneal injections with PBS, 3BrP, gemcitabine or 3BrP combined with gemcitabine for 3 consecutive days, followed by a break of 4 days and a new round of treatment. The administration of 3BrP or gemcitabine alone decreased the tumor growth, but the combination of both agents was superior and completely prevented tumor growth in both, the PANC-1 and the T29-derived tumor xenografts (Fig. 6A, C). No significant effects of the treatment to the body weight of mice were observed (Fig. 6B, D). Immunohistochemistry of tumor tissue sections obtained from PANC-1 xenografts reveals that the single treatments reduced the expression of the ALDH1, CD44, Sox2, CxCR4 and Ki-67, and induced cleavage of Caspase-3, whereas both treatments together had strongest effects (Fig. S4). These data suggest that 3BrP reduces xenograft growth and enhances the therapeutic efficacy of gemcitabine with no obvious side effects.

### 3BrP inhibits the growth and expression of CSC markers in patient-derived primary CSCs

**Figure 7:**
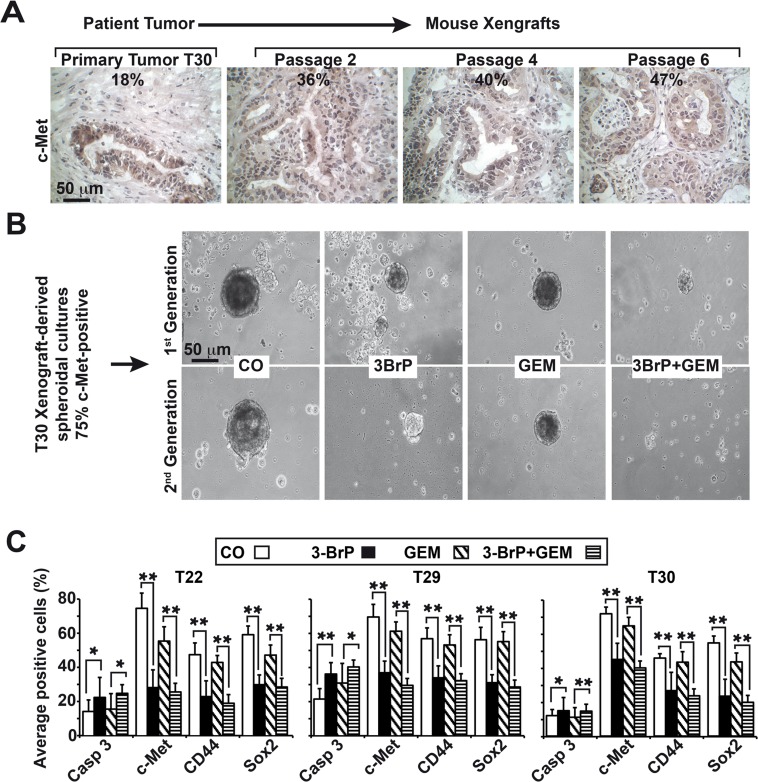
3BrP eliminates primary CSCs derived from patient tissue (A) Patient-derived tissue was transplanted into nude mice, followed by subtransplantation. Representative images of the primary tumor tissue and derived xenografts in passage 2, 4, or 6 stained with c-Met under 400× magnification are shown, and the percentage of c-Met-positive cells is indicated. (B) Anchorage-independent spheroidal cells growing in low-attachment plates were established from xenografts. One week after *in vitro* culture, the spheroids were treated as described in Fig. 2C at a density of 5×10^5^ cells/mL, and photographs were obtained under a microscope (1^st^ Generation). The bar indicates 50 μm. For the secondary spheroids, viable cells were isolated from the primary spheroids after treatment, followed by a second treatment immediately after re-seeding (2^nd^ Generation). (C) Cells from treated primary spheroids were applied to glass slides by cytospin centrifugation. The expression of the cleaved, active fragment of caspase-3, c-Met, CD44, and Sox2 was examined by immunohistochemistry. The number of positive cells per experiment and staining were quantified in at least 10 vision fields under 400× magnification.

To study the effects of 3BrP in primary CSCs, we used the tumor xenograft lines T22, T29, and T30, in which c-Met expression was enriched by serial transplantation of patient tissue on mice to about 50% (Fig. 7A). This percentage was further enriched to 75% c-Met positive cells by growing the isolated tumor cells as anchorage-independent spheres (Fig. 7B, C), which favors the growth of CSCs. The spheres were treated with 3BrP and gemcitabine alone or together. 3BrP strongly diminished the size of spheroids, and gemcitabine had a minor effect. However, the combination treatment was strongest (Fig. 7B) and led to the complete elimination of spheroidal cells in the second-generation spheroids. Immunocytochemistry and analyses of the positive cells revealed that 3BrP significantly induced apoptosis, and inhibited the expression of CSC markers (Fig. 7C, Fig. S5A, B, C). In contrast, gemcitabine had no effect on the expression of CSC markers or apoptosis induction. These data confirm our previous findings and suggest that the inhibition of glucose metabolism by 3BrP prevents stem cell signaling, thereby increasing the effectiveness of gemcitabine.

## DISCUSSION

In our study, we observed the strongly inhibited growth of highly malignant PDA cells upon glucose deprivation, whereas less aggressive PDA cells or normal cells were barely affected. Additionally, colony formation as a feature of the self-renewal potential was strongly inhibited by glucose deprivation or LDH-A inhibition by siRNA transfection. The anti-glycolytic agent 3BrP strongly reduced the viability of the highly malignant PDA cells in a pyruvate- and lactate-dependent manner, but had a reduced effect in less aggressive and gemcitabine-sensitive PDA cells and only marginally affected normal cells. Interestingly, 3BrP is known to inhibit HKII, which mediates the conversion of glucose to glucose-6-phosphate in the first step of glycolysis [16]. This situation, together with the overall effect of 3BrP in impairing cell function by inducing an ATP collapse [15], may have sensitized and eliminated CSC features in our cellular models.

Although no clinical trials, with the exception of a case report, have been documented to date, several 3BrP-based pre-clinical studies with different tumor entities indicate that this alkylating agent is able to overcome therapy resistance [15]. This observation is supported by our study in which we combined 3BrP with gemcitabine, a combination that has not been previously studied. We examined the sensitizing effect by *in vitro* and *in vivo* xenotransplantation studies. A concentration of 50 μM 3BrP was very effective in depleting NAD(P)H and ATP levels in highly malignant PDA cells, as concluded from the reduced viability measured using MTT and Cell Titer Glo assays. Furthermore, the ability to grow as colonies and spheres and the tumor-forming potential *in vivo* was strongly reduced and almost completely abolished with the combination of 3BrP and gemcitabine. This finding is supported by our western blot and NF-κB EMSA results, which showed that 3BrP, but not gemcitabine, strongly inhibited several markers involved in stemness and pluripotency, specifically enhanced NF-κB binding activity, ALDH1 expression and activity and such factors as Notch-1, Oct-4, Sox2, c-Met, CD44, and CxCR4. Our *in vivo* findings that 3BrP inhibited tumor growth, invasion, and metastasis of cells after xenografting to fertilized chicken eggs or mice are in agreement with the results of a recent study. In that study, the tumor progression of PANC-1 xenografts orthotopically transplanted to the mouse pancreas was completely inhibited by the ultrasound-guided intratumoral injection of 3BrP [20], with no observed adverse events. We also did not observe side effects because the weight of the chicken embryos or mice was not diminished significantly and liver necrosis was not detected.

Our data are in accordance with the recent finding that 3BrP disrupts the clonogenic capacity of human KG1 leukemia cells, RPMI8226 myeloma cells, and HepG2 hepatoma cells [23]. The authors of this study suggest that the 3BrP-mediated depletion of ATP inhibits ATP-dependent efflux pumps and thus restores drug sensitivity along with the disruption of clonogenicity. This mechanism might have contributed in our system. Another recent interesting study suggests that 3BrP sensitivity is determined by the expression of the SLC16A1 gene product, mono-carboxylate transporter 1 (MCT1), which is the main determinant of 3BrP uptake by cancer cells [24]. In accordance with our finding of 3BrP sensitivity of MIA-PaCa2 and PANC-1 cells, these two cell lines express MCT1 [25], whereas MCT1 expression to our knowledge was not examined in the other cell lines used in the present study. Given its reactivity as alkylator, 3BrP would be expected to alkylate not only proteins but also DNA and RNA, similar to e.g. cisplatin, No such DNA damage or DNA/RNA alkylation have been reported, however [26]. In line with this assumption, a case control study [21] and our own data suggest that the side effects of 3BrP are tolerable.

In conclusion, our findings extend the knowledge about the remarkable anti-cancer properties of 3BrP against CSCs and there is an urgent need to bring 3BrP to clinical trials.

## METHODS

### Human primary and established pancreatic cancer cell lines

The human established PDA lines MIA-PaCa2, PANC-1, AsPC-1, BxPc-3, and Capan-2 and human CRL 4023 hTERT-HPNE immortalized pancreatic ductal cells were obtained from American Type Culture Collection (Manassas, VA, USA). Human primary pancreatic stellate cells were kindly provided by Dr. O. Strobel (General Surgery, University of Heidelberg, Germany), and human primary skin fibroblasts were kindly provided by Dr. H.-J. Stark (DKFZ, Heidelberg, Germany). The established cell lines were regularly cultured in high-glucose DMEM (18 mmol/L glucose) supplemented with 10% FCS and 5% HEPES. The CRL 4023 cells were cultured in ATCC complete growth medium. Primary PDA cell lines PaCa DD 183, PaCa DD 159, and PaCa DD 135 were isolated and cultured as described [27]. The established cell lines were recently authenticated by a commercial service (Multiplexion, Heidelberg, Germany). Mycoplasma-negative cultures were ensured by monthly mycoplasma tests.

### Reagents

3BrP (bromopyruvic acid >97% pure, Sigma-Aldrich Chemie GmbH, Steinheim, Germany), 2-deoxy-d-glucose (2-DG, Sigma-Aldrich), Bortezomib (B, Selleck Chemicals, Houston, TX, USA) were freshly dissolved in sterile PBS. Sodium lactate (>99% pure, Sigma-Aldrich) and sodium pyruvate (>99% pure Sigma-Aldrich) were freshly dissolved in the cell culture medium. A gemcitabine solution (126 mM, Eli Lilly, Indianapolis, IN, USA) was diluted in the cell culture medium (100 μM stock). The final concentrations of the solvents in media were 0.1% or less.

### Selection of CSC-enriched spheroidal cells from patient tumors

Surgical, non-diagnostic specimens were transplanted into NMRI (nu/nu) mice followed by subtransplantation and ex vivo spheroidal culture as described [28, 29]. The patient material was obtained under the approval of the ethics committee of the University of Heidelberg after written informed consent was obtained from the patients. The diagnoses were established by conventional clinical and histological criteria according to the World Health Organization (WHO). All surgical resections were indicated by the principles and practice of oncological therapy.

### Measurement of glucose consumption and lactate production

The extracellular glucose and lactate concentrations in the culture supernatants were measured using a DRY-CHEM FDC3500 machine (Fuji (Medical Co. Ltd., Tokyo, Japan) in the diagnostic laboratory of the University Clinic Heidelberg.

### Cell viability assays

Cell viability was measured using 3-(4,5-dimethylthiazol-2-yl)-2,5-diphenyltetrazolium bromide (MTT), as described previously [7], or using the CellTiter-Glo Luminescent Cell Viability Assay, as described by the manufacturer (Promega GmbH, Mannheim, Germany).

### Annexin V staining and FACS analysis of apoptosis

The cells were treated with FITC-conjugated annexin-V (Life Technologies GmbH, Darmstadt, Germany) and analyzed by FACS, as previously described [30].

### Detection of ALDH1 activity

The ALDEFLUOR substrate (Aldagen, Inc., Durham, NC, USA) was added to the tumor cells, and the ALDH1 activity was measured by FACS analysis according to the instructions of the manufacturer.

### Colony and spheroid forming assay

Colony and spheroid forming assays were performed as described previously [7].

### Western blot analysis

Whole-cell extracts were prepared using a standard protocol [7], and the proteins were detected by western blotting. The following antibodies were used: mouse mAbs against human ALDH1 (BD Biosciences, Germany), Oct-4 (Cell Signaling Technology, MA, USA), and GLUT-1 (Thermo Scientific Inc., Germany); rabbit mAbs against Notch1, Sox2, and LDH-A (Cell Signaling Technology, MA, USA); rabbit polyclonal Ab against LDH-B (Thermo Fisher Scientific Pierce, Bonn, Germany).

### siRNA transfection targeting LDH1

Specific FlexiTube GeneSolution siRNAs against LDH-A or LDH-B were obtained from Qiagen (MD, USA), and the Mission siRNA Universal Negative Control was purchased from Sigma-Aldrich (St. Louis, MO, USA). Transfections were performed using the Lipofectamine™ RNAiMAX kit from Invitrogen (Carlsbad, CA; USA) according to the manufacturer's instructions.

### Electrophoretic mobility shift assay (EMSA) of NF-κB binding

Nuclear protein extracts were prepared using the NE-PER® Nuclear and Cytoplasmic Extraction Reagents, and the bandshift reaction was performed as we described recently [31].

### Transplantation of tumor cells to the CAM of fertilized chicken eggs

This assay was performed as we described recently [32]. Tumor volumes were estimated using the following formula: Volume = 4/3 × Π × r^3^ (r = 1/2 × √ of diameter 1 × diameter 2).

### Subcutaneous transplantation of PDA cells into mice

PANC-1 or T29 cells (1 × 10^6^) were transplanted in 50% matrigel/PBS *s.c.* to the left and right flanks of five-week-old female NMRI-nu immunodeficient mice (Janvier Labs, France) in a total volume of 100 μl. The tumor sizes were measured and analyzed as described previously [7]. The animal experiments were carried out in the animal facilities of the University of Heidelberg after approval by the authorities (Regierungspräsidium Karlsruhe, Germany).

### Immunohistochemistry and immunofluorescence staining

Staining was performed on 6-μm frozen tissue sections or on spheroidal cultures as we described previously [29]. The primary antibodies were: Rabbit pAbs against human c-Met, CD133, and Sox2 (Abcam, Cambridge, UK), CD44 (GeneTex Inc., USA); E-cadherin (Cell Signaling, Danvers, MA, USA); Ki67 (Thermo Scientific, Rockford, IL, USA); the cleaved fragment of activated human caspase-3 (R&D Systems, Abingdon, UK); c-Rel (Santa Cruz Biotechnology Inc., Germany); ALDH1 (BD Transduction Laboratories, USA); and CxCR4 (GeneTex Inc., USA); mouse mAb anti-cytokeratin 19 was from Abcam.

### Statistical analysis

The data obtained using the established and primary cell lines, excluding both the antibody protein arrays and patient xenograft-derived spheroidal cells, are presented as the mean ± SD of at least three separate experiments performed in triplicate. The statistical significance was evaluated by Student's t-test with a Bonferroni correction (*p<0.05, **p<0.01). The experiments with the primary spheroidal cells were performed twice, and at least 10 fields of view per staining were counted. The statistical significance of a total number of at least 20 fields of view was evaluated by Student's t-test, and an analysis of variance was performed.

## SUPPLEMENTARY MATERIAL AND FIGURES



## Abbreviations

Aldehyde dehydrogenase 1 (ALDH1)
Cancer stem cells (CSCs)
Chorioallantoic membrane (CAM)
Electrophoretic mobility shift assay (EMSA)
Pancreatic ductal adenocarcinoma (PDA)
