# Supplementary material for: Inhibition of glucose turnover by 3-bromopyruvate counteracts pancreatic cancer stem cell features and sensitizes cells to gemcitabine
Source: Oncotarget. 2014 Jun 15;5(13):5177–89. doi: 10.18632/oncotarget.2120 (PMC4148131; doi:10.18632/oncotarget.2120)
Supplement: Supplementary file 1 [file oncotarget-05-5177-s001.pdf]

Inhibition of glucose turnover by 3-bromopyruvate counteracts pancreatic cancer stem cell features and sensitizes cells to gemcitabine

Supplementary Material

CSC-Properties		Less aggressive		Highly aggressive			References
		BxPc-3	Capan2	AsPC-1	MIA-PaCa2	PANC-1	
Source		Primary tumor	Primary tumor	Ascites	Primary tumor	Primar tumor	ATCC
Tumor Grade		G2	G1	G2	G3	G3	(33)
p53 status		MT	WT	MT	MT	MT	(33)
K-ras status		WT	MT	MT	MT	MT	(33)
In vitro morphology		Densely attached	Densely attached	Loosely attached	Loosely attached	Loosely attached	ATCC, present study
Self Renewal Capacity	Colony-formation	+	+	+++	+++	+++	(7), present study
	Spheroid formation	-	-	+	+++	+++	(7), present study
	ALDH1 activity	+	+	++	+++	+	(7), present study
Gemcitabine resistance		+	++	+++	+++	+++	(34), present study
E-Cadherin		+++	++	+	-	+	(35, 36)
Vimentin		+	-	+++	+++	+++	(35-38)

None (-); Weak (+); Median (++); Strong (+++); MT (mutant), WT (wild-type).

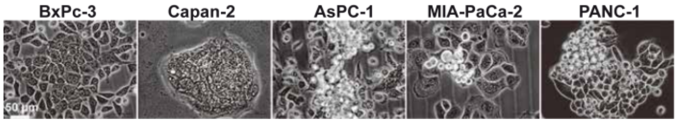

Supplementary figure1: **Summary of CSC features in established cell lines used and morphology.**

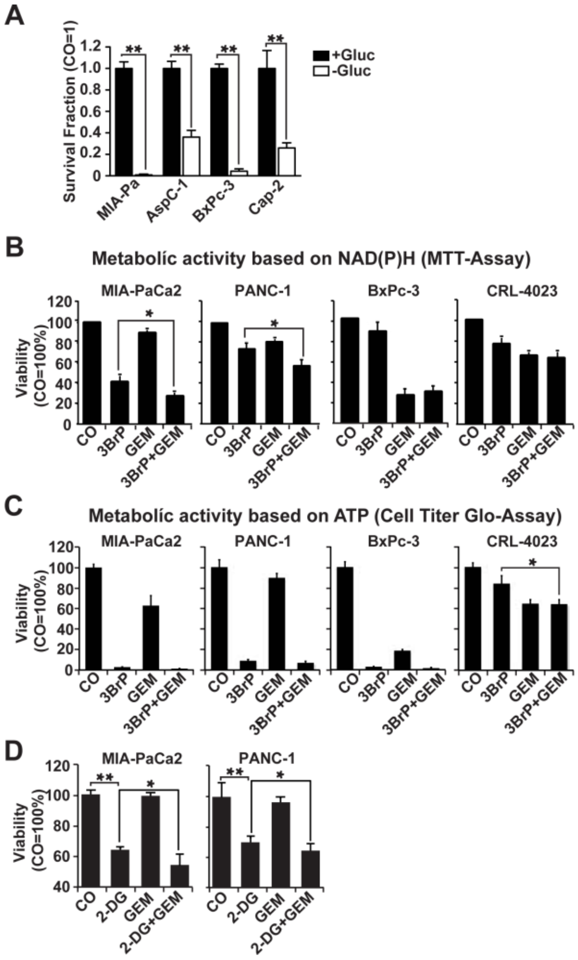

Supplementary figure2: Inhibition of glycolysis inhibits colony formation and viability in highly malignant cells and sensitizes them to gemcitabine. (A) The highly malignant MIA-PaCa2 and PANC-1 cells and the less malignant BxPc-3 and Capan-2 cells were seeded at a density of 3×105 cells in 6-well tissue culture plates and cultured in a medium with high glucose (25 mmol/L). Seventy-two hours later the cells were trypsinized and 200 MIA-PaCa2 and AsPC-1 or 2000 BxPc-3 or Capan-2 cells/well were seeded in 6-well plates in the presence of a medium with a minimal glucose amount (0.15 mmol/L), or in medium with a high glucose amount (25 mmol/L). Cells were grown without a change of the medium for two weeks, followed by evaluation of fixed and Coomassie-stained colonies consisting of at least 50 cells. The plating efficiency as a percent was calculated using the following formula: 100 × number of colonies/number of seeded cells. (B) MIA-PaCa2, PANC-1 and BxPc-3 cells and the non-malignant immortalized pancreatic ductal CRL 4023 cells, were treated as described in Fig. 2C; metabolic activity based on NAD(P)H turnover, reflecting viability, was measured by an MTT assay. The control was set to 100%. (C) Cells were treated as described above, and the metabolic activity based on ATP turnover, reflecting viability, was measured with the Cell Titer Glo Assay and analyzed as described above. (D) Cells were pre-treated with 2-deoxy-d-glucose (2-DG, 10 mM). Twenty-four hours later cells were treated with gemcitabine (50 nM, GEM) for additional 72 h - either alone or combined with 2-DG as indicated.

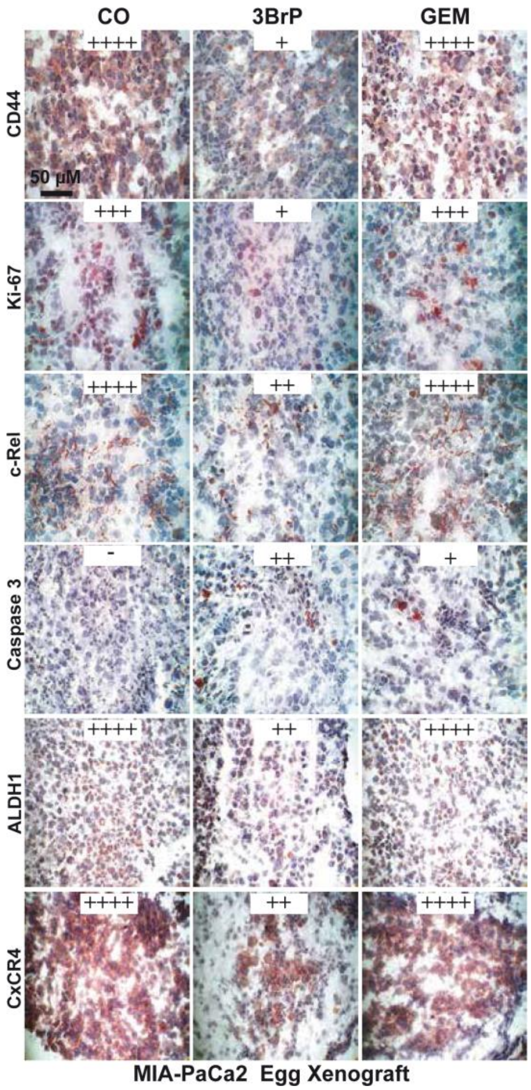

Supplementary figure3: 3BrP inhibits tumor growth and downregulates CSC markers in human PDA xenografts transplanted to chicken eggs. Frozen xenograft tumor tissue sections derived from the experiment shown in Fig. 5A were analyzed by immunohistochemistry for the expression of the CSC markers ALDH1, CD44, and Sox2, the invasion marker CxCR4, the proliferation marker Ki-67, the NF-κB subunit c-Rel, and the apoptosis marker "cleaved fragment of active caspase 3". Representative photographs under 400× magnification are shown. Very high (++++), high (+++), medium (++), low (+), and very low to absent (-) expression is indicated.

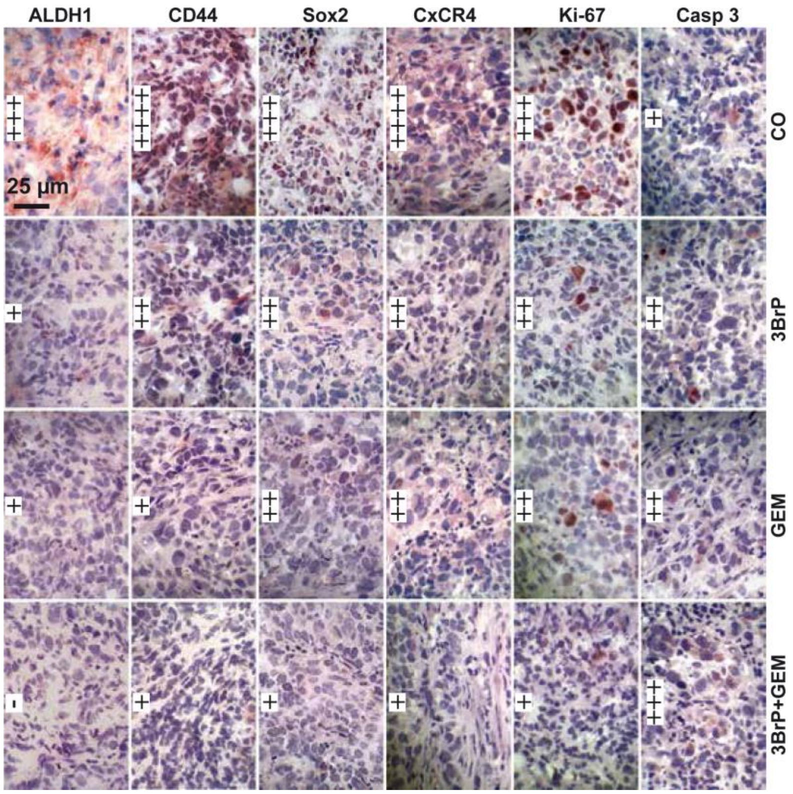

Supplementary figure4: 3BrP inhibits tumor growth and downregulates CSC markers in human PDA xenografts transplanted into mice. Frozen mouse xenograft tumor sections from transplanted PANC-1 cells were analyzed as described in Fig. 5 F.

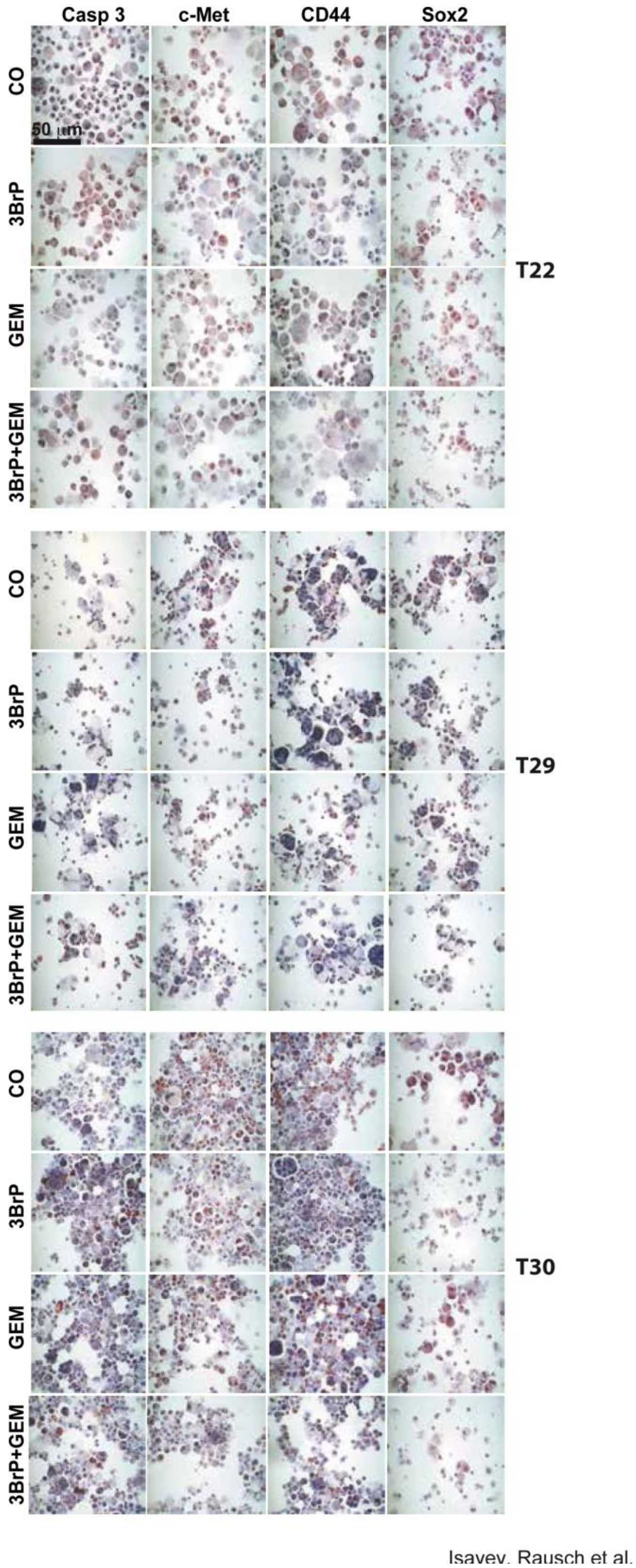

Supplementary figure5: 3BrP inhibits tumor growth and downregulates CSC markers *in vivo.* (C) CSC-enriched spheroidal cultures were isolated, treated and analyzed as described in Fig. 7; Representative stainings of the immunohistochemistry detection of the cleaved, active fragment of caspase 3, c-Met, CD44, and Sox2 in T22, T29, and T30 are shown. The bar indicates 20 µm.
